# Targeting glutamine metabolism sensitizes pancreatic cancer to PARP-driven metabolic catastrophe induced by ß-lapachone

**DOI:** 10.1186/s40170-015-0137-1

**Published:** 2015-10-12

**Authors:** Gaurab Chakrabarti, Zachary R. Moore, Xiuquan Luo, Mariya Ilcheva, Aktar Ali, Mahesh Padanad, Yunyun Zhou, Yang Xie, Sandeep Burma, Pier P. Scaglioni, Lewis C. Cantley, Ralph J. DeBerardinis, Alec C. Kimmelman, Costas A. Lyssiotis, David A. Boothman

**Affiliations:** Department of Pharmacology, University of Texas Southwestern Medical Center, 6001 Forest Park Drive, Dallas, 75390-8807 TX USA; Radiation Oncology, University of Texas Southwestern Medical Center, Dallas, TX USA; Touchstone Diabetes Center, Simmons Comprehensive Cancer Center, University of Texas Southwestern Medical Center, Dallas, TX USA; Department of Internal Medicine, Weill Cornell Medical College, 413 East 69th Street, BB-1362, New York, NY 10021 USA; Department of Bioinformatics and Biostatistics, Clinical Sciences, UT Southwestern Medical Center, 5323 Harry Hines Boulevard, Dallas, TX 75390 USA; Department of Medicine, Weill Cornell Medical College, 413 East 69th Street, BB-1362, New York, NY 10021 USA; Children’s Medical Center Research Institute, UT Southwestern Medical Center, 5323 Harry Hines Blvd, Dallas, TX 75390 USA; Department of Radiation Oncology, Division of Genomic Stability and DNA Repair, Dana-Farber Cancer Institute, Boston, MA 02215 USA; Department of Molecular and Integrative Physiology, University of Michigan, Ann Arbor, MI 48109 USA; Internal Medicine, Division of Gastroenterology, University of Michigan, Ann Arbor, MI 48109 USA

**Keywords:** Metabolic cancer therapy, Glutamine metabolism, Transamination, NQO1-bioactivated drugs

## Abstract

**Background:**

Pancreatic ductal adenocarcinomas (PDA) activate a glutamine-dependent pathway of cytosolic nicotinamide adenine dinucleotide phosphate (NADPH) production to maintain redox homeostasis and support proliferation. Enzymes involved in this pathway (*GLS1* (mitochondrial glutaminase 1), *GOT1* (cytoplasmic glutamate oxaloacetate transaminase 1), and *GOT2* (mitochondrial glutamate oxaloacetate transaminase 2)) are highly upregulated in PDA, and among these, inhibitors of *GLS1* were recently deployed in clinical trials to target anabolic glutamine metabolism. However, single-agent inhibition of this pathway is cytostatic and unlikely to provide durable benefit in controlling advanced disease.

**Results:**

Here, we report that reducing NADPH pools by genetically or pharmacologically (bis-2-(5-phenylacetamido-1,2,4-thiadiazol-2-yl)ethyl sulfide (BPTES) or CB-839) inhibiting glutamine metabolism in mutant Kirsten rat sarcoma viral oncogene homolog (*KRAS*) PDA sensitizes cell lines and tumors to ß-lapachone (ß-lap, clinical form ARQ761). ß-Lap is an NADPH:quinone oxidoreductase (*NQO1*)-bioactivatable drug that leads to NADPH depletion through high levels of reactive oxygen species (ROS) from the futile redox cycling of the drug and subsequently nicotinamide adenine dinucleotide (NAD)+ depletion through poly(ADP ribose) polymerase (*PARP*) hyperactivation. *NQO1* expression is highly activated by mutant *KRAS* signaling. As such, ß-lap treatment concurrent with inhibition of glutamine metabolism in mutant *KRAS*, *NQO1* overexpressing PDA leads to massive redox imbalance, extensive DNA damage, rapid *PARP*-mediated NAD+ consumption, and PDA cell death—features not observed in *NQO1*-low, wild-type *KRAS* expressing cells.

**Conclusions:**

This treatment strategy illustrates proof of principle that simultaneously decreasing glutamine metabolism-dependent tumor anti-oxidant defenses and inducing supra-physiological ROS formation are tumoricidal and that this rationally designed combination strategy lowers the required doses of both agents *in vitro* and *in vivo*. The non-overlapping specificities of *GLS1* inhibitors and ß-lap for PDA tumors afford high tumor selectivity, while sparing normal tissue.

**Electronic supplementary material:**

The online version of this article (doi:10.1186/s40170-015-0137-1) contains supplementary material, which is available to authorized users.

## Background

**Fig. 1:**
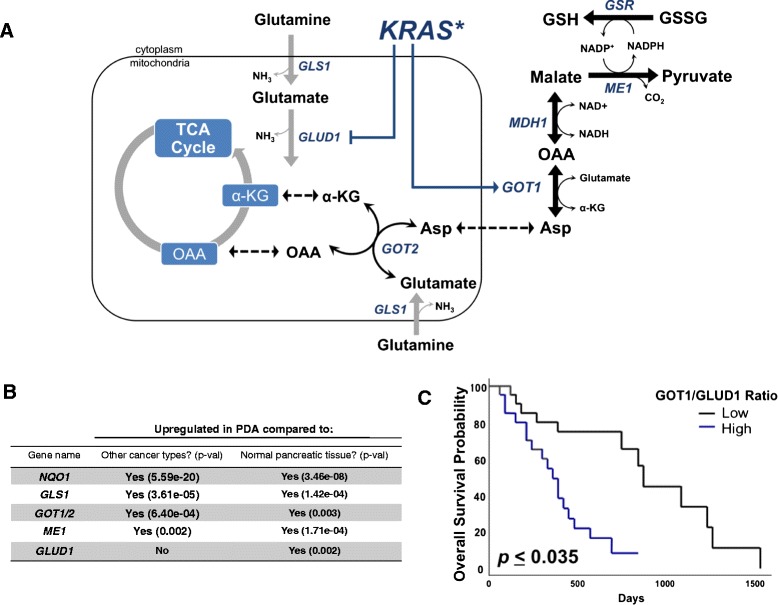
Glutamine metabolism genes are upregulated in PDA. **a** Glutamine utilization pathway in PDA. *Asp* aspartate, *GSR* glutathione-disulfide reductase. **b** *NQO1* and glutamine metabolism enzymes assessed in patient tumor tissue in PDA versus 17 other cancer types (*central columns*, “other” cancers include solid and hematological malignancies) and PDA versus normal pancreatic tissue (*right most column*). Data were obtained from Oncomine (www.oncomine.org). **c** Kaplan–Meier survival curve corrected for multiple comparisons of 45 PDA patients grouped according to high versus low *GOT1:GLUD1* expression assessed using the PROGgene webtool (http://watson.compbio.iupui.edu/chirayu/proggene/database/?url=proggene)

Pancreatic ductal adenocarcinoma (PDA) is a recalcitrant cancer in which patients have <6 % 5-year survival rates. Mortality from this disease is predicted to be the second leading cause of cancer-related death by 2020 [1]. PDAs are highly resistant to conventional chemotherapies [1], and activating mutations in Kirsten rat sarcoma viral oncogene homolog (*KRAS*) are present in >95 % of all cases [2]. Despite significant effort from the pharmaceutical industry over the past 30 years, *KRAS* has proven to be a challenging drug target [3]. An emerging therapeutic approach is to target alterations in PDA metabolism driven by mutant *KRAS* [2, 4–6]. For example, PDA cells generate the bulk of the ribose used for de novo nucleotide biosynthesis through the non-oxidative arm of the pentose phosphate pathway [7]. This *KRAS*-driven reprogramming of glucose metabolism bypasses the nicotinamide adenine dinucleotide phosphate (NADPH)-generating oxidative arm. To compensate for this rewiring, PDAs utilize glutamine through a *GLS1* (mitochondrial glutaminase)-, *GOT2* (mitochondrial glutamate oxaloacetate transaminase 2)-, and *GOT1* (cytoplasmic glutamate oxaloacetate transaminase 1)-dependent pathway to support cellular redox balance in the face of rapid proliferation and growth (Fig. 1a) [2, 8, 9]. This is in contrast to the canonical metabolism of glutamine-derived glutamate through *GLUD1* (glutamate dehydrogenase 1) to supply carbon backbone to the TCA cycle. Genetic inhibition of enzymes in this pathway is profoundly growth inhibitory in PDA but does not result in the induction of a cytotoxic response. These results suggest that a means to induce redox balance in PDA, concurrent with inhibition of this *KRAS*-dependent Gln metabolism pathway, may provide a means to induce tumor-selective killing.

In an attempt to leverage increased tumor-cell reliance on glutamine, small molecule inhibitors of *GLS1* were developed (e.g., bis-2-(5-phenylacetamido-1,2,4-thiadiazol-2-yl)ethyl sulfide (BPTES), CB-839, compound 968) [10–12]. *GLS1* catalyzes the first step in the PDA glutamine metabolism pathway, converting glutamine to glutamate (Fig. 1a) [8]. As such, *GLS1* inhibition in PDA cells in culture leads to a block in glutamine metabolism but, as with the genetic approaches above, lacks cytotoxicity. Moreover, while *GLS1* inhibitors are potent inhibitors of cell proliferation in cell culture models, they have relatively minor effects on tumor growth in pre-clinical cancer models as single agents [13–17].

To increase the specificity and efficacy of *GLS1* inhibition in PDA, we combined BPTES or CB-839 with ß-lapachone (ß-lap), a targeted cancer therapeutic that causes tumor-selective reactive oxygen species (ROS) formation in an NADPH:quinone oxidoreductase 1 (*NQO1*)-specific manner [18]. *NQO1* is highly expressed in many types of cancer, including PDA. In fact, elevated *NQO1* expression (≥tenfold) has been observed in ~90 % of PDA patient specimens, making PDA an especially appealing target for therapy using *NQO1*-bioactivatable drugs, such as ß-lap [18–21].

ß-Lap is a substrate for two-electron oxidoreduction by *NQO1*, an inducible phase II quinone-detoxifying enzyme. The resulting hydroquinone form of ß-lap is highly unstable and spontaneously reacts with oxygen to revert back to the parent compound, generating two moles of superoxide per mole NAD(P)H used in the process [18]. This leads to a futile cycle that occurs rapidly in *NQO1*-overexpressing cells resulting in massive ROS formation, oxidative DNA damage, and H_2_O_2_-mediated DNA single-strand breaks (Additional file 1: Figure S1A). In an attempt to repair this damage, poly(ADP ribose) polymerase (*PARP*) becomes hyperactivated, generating extensive free branched poly(ADP ribose) (PAR) polymer levels. The hyperactivated *PARP* substantially depletes intracellular nicotinamide adenine dinucleotide (NAD)+ and adenosine triphosphate (ATP) pools and ultimately overwhelms the ability of the DNA repair machinery to repair ß-lap-induced DNA lesions.

The therapeutic window provided by *NQO1* expression (and thus *NQO1*-mediated bioactivation of ß-lap) has advanced ß-lap to phase I and Ib clinical trials (ARQ761) [22]. Unfortunately, dose-limiting methemoglobinemia caused by nonspecific ROS generation at high ß-lap doses somewhat limits the efficacy of ß-lap as monotherapy [22]. Strategies for increasing cancer cell cytotoxicity, while maintaining *NQO1* specificity, could further enhance efficacy of ß-lap for therapy against PDAs.

ß-Lap and *GLS1* inhibition have distinct but highly complementary mechanisms of action. ß-Lap induces tumor-selective ROS generation specifically in PDA cells that express high levels of *NQO1*. *GLS1* inhibition primes PDA cancer cells for death by lowering anti-oxidant pools derived from glutamine, sensitizing the cell to ROS damage. Here we show, using an in vivo pre-clinical model of PDA, that the increased dependence of PDA cells on glutamine is specifically targeted by exposure to both drugs. The use of ß-lap with *GLS1* inhibitors results in synergistic *NQO1*- and *PARP*-dependent cancer cell death, allowing use of lower doses and shorter treatment times for both agents.

## Results

### Glutamine metabolism genes are upregulated in PDA

Enzymes utilized for glutamine metabolism in PDA, *GLS1*, *GOT1/2*, and malic enzyme 1 (*ME1*) (see pathway, Fig. 1a), in addition to *NQO1*, were significantly upregulated in PDA compared to 17 other cancers when assessed using the Oncomine webtool (Fig. 1b) [8]. This was apparent in both cell lines and tumor samples. In contrast, *GLUD1*, which diverts glutamine carbon away from the *GOT2–GOT1–ME1* pathway into an alternate metabolic pathway, was not upregulated in PDA relative to other cancer types (Fig. 1a). Additionally, glutamine metabolic enzymes, *NQO1*, and *GLUD1* were found to be significantly upregulated in PDA relative to normal pancreatic tissue (Fig. 1a). To determine the clinical relevance associated with the PDA glutamine metabolic pathway relative to other enzymes involved in glutamine metabolism, we evaluated the association of individual gene expression levels with overall survival in the data set that contained clinical follow-up information [23]. Kaplan–Meier analysis did not show a significant difference in outcome when patients were separated into high and low expression levels of the genes of interest (data not shown). However, when *GOT1* and *GOT2* were normalized to *GLUD1* expression, we found that a high *GOT1* to *GLUD1* or *GOT2* to *GLUD1* ratio was significantly associated with poor outcome (Fig. 1c, Additional file 1: Figure S1B). These data suggest that gene expression of *GOT* isoforms are generally elevated in PDA and have prognostic and functional significance relative to *GLUD1* expression.

### Inhibiting glutamine metabolism sensitizes PDA to ß-lap

**Fig. 2:**
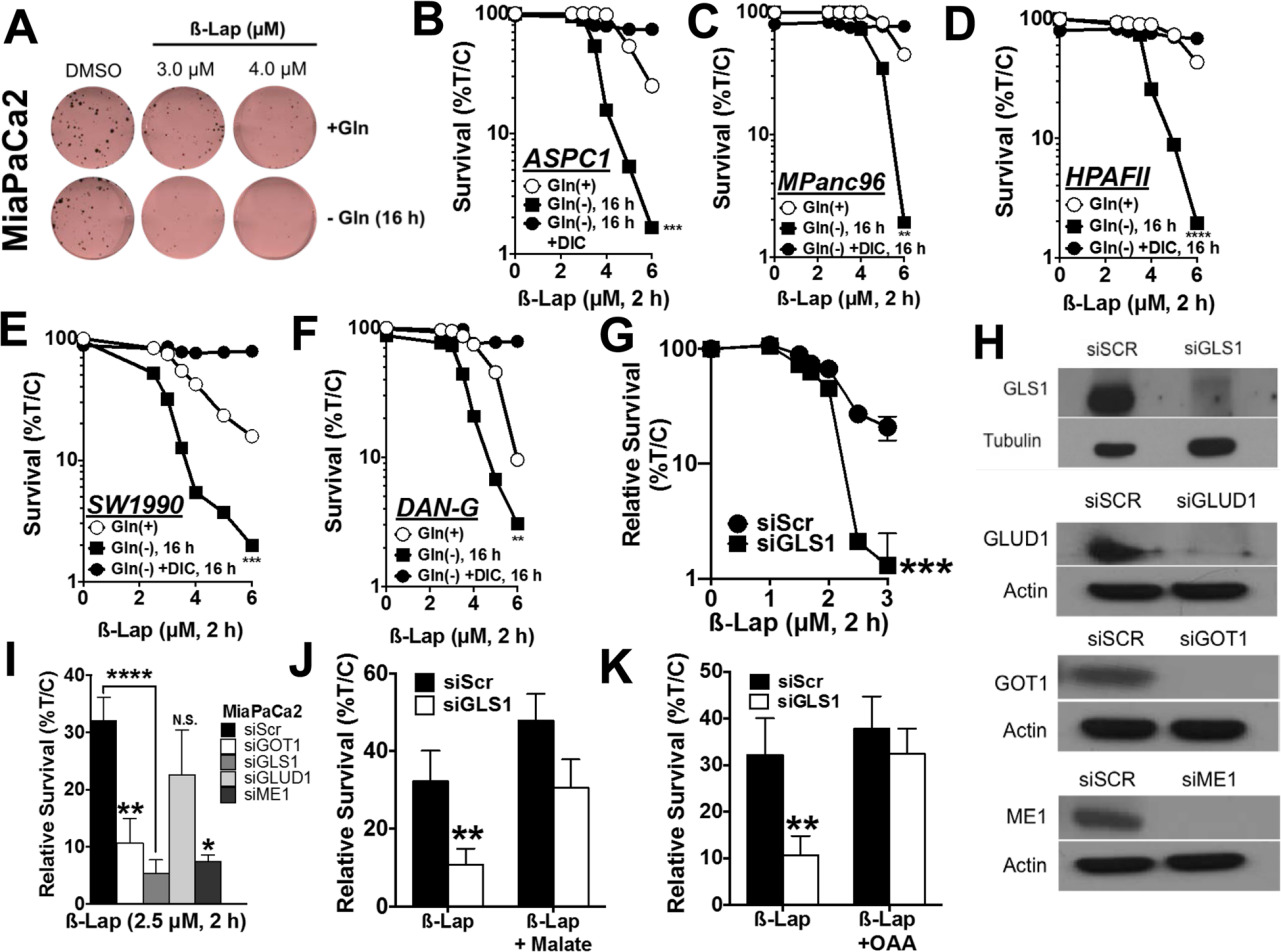
Inhibiting glutamine metabolism sensitizes PDA to ß-lap. **a** Colony formation assay of MiaPaCa2 cells depleted of glutamine for 16 h, followed by treatment with ß-lap for 2 h. *p < 0.01* at 3 μM and *p < 0.0001* at 4 μM. **b**–**f** Glutamine deprivation experiments with a dose range of ß-lap in ASPC1, MPanc96, HPAFII, SW1990, and DAN-G PDA cell lines. **g** ß-lap dose response in MiaPaCa2 cells with knockdown of *GLS1*. **h** Western blots for *GLS1*, *ME1*, *GOT1*, and *GLUD1* upon knockdown in MiaPaCa2 cell lines for 48 h using siRNA. **i** Relative survival of *GOT1*, *GLS1*, *GLUD1*, and *ME1* MiaPaCa2 knockdown cells treated with 2.5 μM ß-lap for 2 h. **j**, **k** *GLS1* was knocked down in MiaPaCa2, and either 3 mM oxaloacetate (OAA) or 3 mM dimethyl malate was added for 24 h followed by a 2-h treatment with 2.5 μM ß-lap. *Relative survival* represents means of CellTiter-Glo survival assay 48 h after treatment plotted as percentage treated/control (T/C), ±SE from sextuplicate samples. All results were compared using Student’s *t* tests. *p < 0.05; **p < 0.01; ***p < 0.001; ****p < 0.0001

Given the reliance of PDA on glutamine metabolism for redox balance, we hypothesized that glutamine deprivation of *NQO1*-overexpressing PDA cells would sensitize them to ß-lap exposure by lowering anti-oxidant defenses and increasing *NQO1*-induced ROS damage. MiaPaCa2 cells were grown in Gln-free or Gln-containing (2 mM) media for 16 h and then exposed to ß-lap for 2 h. Short-term Gln deprivation did not significantly alter clonogenic survival on its own (Fig. 2a) but did sensitize MiaPaCa2 cells to ß-lap at sub-lethal and higher doses of the drug (Fig. 2a). To confirm these results, we repeated this experiment in five other PDA cell lines: ASPC1, MPanc96, HPAFII, SW1990, and DAN-G (Fig. 2b–f). Additionally, we demonstrated that the observed cytotoxicity was NQO1-dependent, and as addition of the potent *NQO1* inhibitor, dicoumarol (DIC), spared cells from lethality (Fig. 2b–f) [24–26].

Next, RNAi-mediated knockdown of glutamine metabolism enzymes revealed that *GLS1*, *GOT1*, and *ME1* dramatically sensitized MiaPaCa2 and ASPC1 PDA cell lines to ß-lap, relative to non-targeting control (scramble small interfering RNA (siScr)) (Fig. 2g–k). Consistent with the mechanism by which glutamine is metabolized in PDA to maintain redox balance [8], knockdown of *GLUD1* had no effect on ß-lap sensitivity (Fig. 2i). Sensitization of ß-lap-treated MiaPaCa2 cells by *GLS1* knockdown was rescued by replenishing metabolic substrates of the glutamine metabolism pathway that are downstream of the *GLS1* reaction, namely, oxaloacetate (OAA) or cell-permeable dimethyl malate (Fig. 2j, k). These data indicate that PDA cells have an increased reliance on glutamine to generate NADPH (Fig. 1a) in the presence of ß-lap-induced ROS stress.

### *GLS1* inhibition by BPTES sensitizes PDA to ß-lap in an *NQO1*-dependent manner

**Fig. 3:**
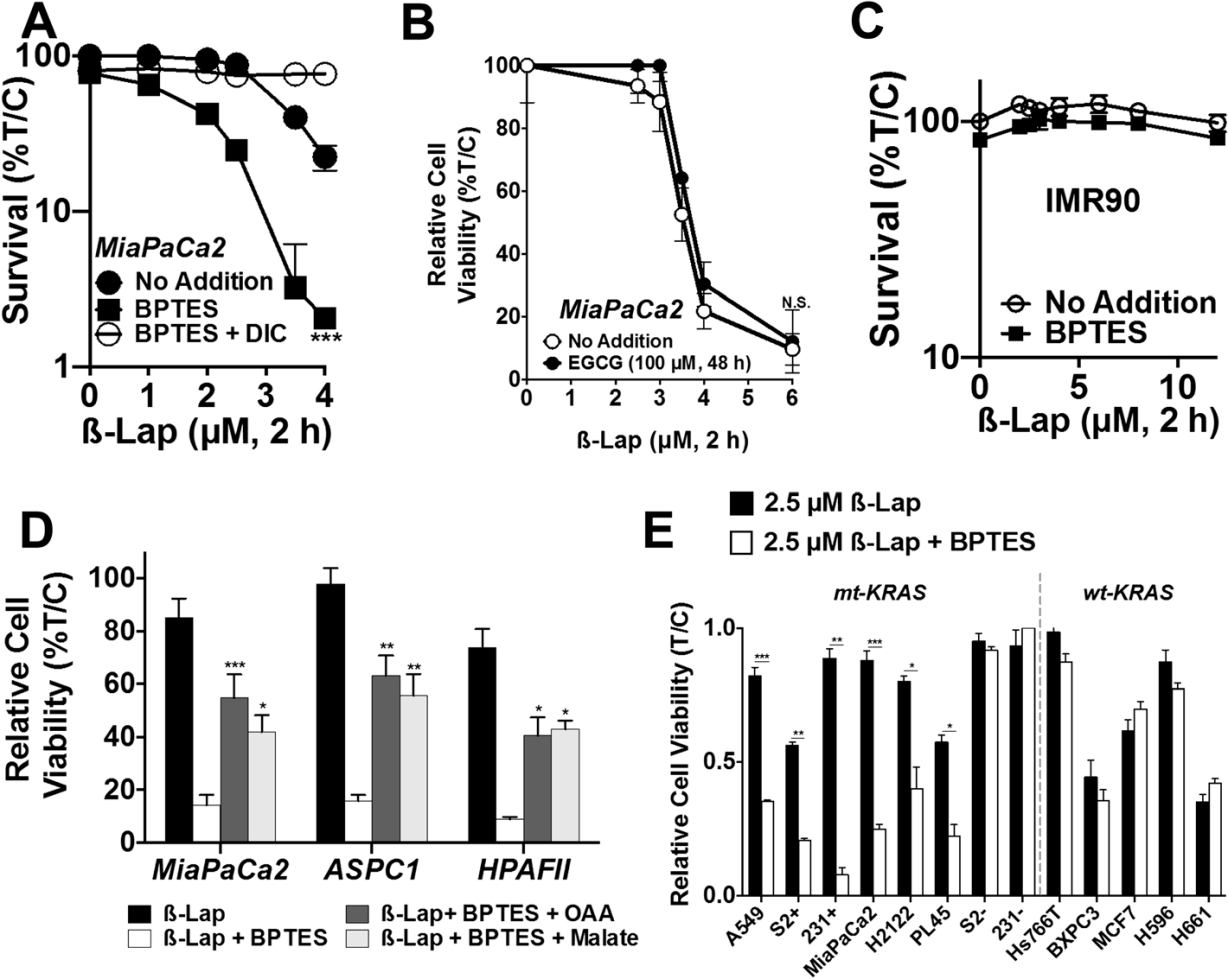
*GLS1* inhibition by BPTES sensitizes PDA to ß-lap in an *NQO1*-dependent manner. **a** Clonogenic survival assay of MiaPaCa2 pre-treated ± 500 nM BPTES for 48 h followed by the addition of ß-lap ±50 μM for 2 h. Data represent survival means ± SE from quadruplicate samples. **b** MiaPaCa2 cells treated with 100 μM of the *GLUD1* inhibitor, EGCG, for 48 h followed by 2-h ß-lap dose response. Relative cell viability represents mean of CellTiter-Glo survival assay 48 h after ß-lap treatment plotted as percentage treated/control (T/C) ± SE from sextuplicate samples. **c** Clonogenic survival assay of normal lung fibroblast cell line, IMR90, pre-treated with ±500 nM BPTES for 48 h followed by 2 h of ß-lap treatment. Data represent survival means ± SE from quadruplicate samples. **d** MiaPaCa2, ASPC1, and HPAFII PDA cell lines were pre-treated with ± 500 nM BPTES for 48 h, either 3 mM oxaloacetate (OAA) or 3 mM dimethyl malate for the last 24 h, and 2.5 μM ß-lap for 2 h. Relative cell viability represents means of CellTiter-Glo survival assay 48 h after treatment plotted as percentage (T/C) ± SE from sextuplicate samples. **e** Various cancer cell lines pre-treated with ± 500 nM BPTES (sub-growth inhibitory) for 48 h followed by the addition of 2.5 μM ß-lap for 2 h. Mutant (mt) *KRAS* lines: A549 non-small cell lung (*NSCL*), PL45 PDA, *NQO1* expressing S2-013 (*S2+*) PDA, *NQO1* expressing MDA-MB-231 (*231+*) triple-negative breast, H2122 NSCL, *NQO1*-deficient S2-013 (*S2−*) PDA, and *NQO1* deficient MDA-MB-231 (*231−*) triple-negative breast cancer cells. Wild-type (wt) *KRAS* lines: *Hs766T* PDA, *BxPC3* PDA, *MCF7* breast, as well as *NQO1+ H596* NSCL and *H661* NSCL cancer cell lines

To pharmacologically replicate the ß-lap sensitization to inhibition of Gln metabolism, MiaPaCa2 cells were treated with a sub-lethal dose of the mitochondrial *GLS1* inhibitor, BPTES (500 nM, 48 h, Additional file 2: Figure S2) and then exposed to various doses of ß-lap for 2 h, with or without DIC (Fig. 3a). BPTES pre-treatment in combination with ß-lap significantly reduced clonogenic survival versus ß-lap alone, while addition of DIC spared the lethality (Fig. 3a). To confirm that our results were due to inhibition of the glutamine-dependent transamination pathway and not the alternative glutamine metabolism pathway through *GLUD1* , we pre-treated MiaPaCa2 cells with epigallocatechin gallate (EGCG), an inhibitor of *GLUD1*  [27] for 48 h and then exposed them to ß-lap. Consistent with our RNAi results (Fig. 2i), we found that *GLUD1* inhibition by EGCG had no effect on ß-lap sensitivity (Fig. 3b). Furthermore, normal human IMR-90 embryonic lung fibroblasts, which have low *NQO1* levels [26] were not affected by ß-lap, with or without BPTES treatments (Fig. 3c). Replenishing the NADPH-producing transamination pathway with the addition of OAA or dimethyl malate, metabolites downstream of *GLS1*, rescued BPTES-dependent hypersensitivity to ß-lap in MiaPaCa2, ASPC1, and HPAFII PDA cells (Fig. 3d).

Mutant *KRAS* signaling drives *NQO1* expression and glutamine dependence [7, 8, 28, 29], thus we sought to assess the generality of mutant *KRAS* expression on the effects of BPTES and ß-lap. BPTES pre-treatment sensitized a variety of *NQO1*-expressing *KRAS* mutant cancer cell lines to ß-lap, including lung, triple-negative breast, and additional PDA cell lines. In contrast, *NQO1*-deficient *KRAS* mutant lines remained resistant to ß-lap, whether or not BPTES was added (Fig. 3e). In addition, pre-treatment with 500 nM BPTES for 48 h did not increase the sensitivities of ß-lap-responsive, *NQO1*-expressing *KRAS* wild-type lung, breast, or pancreatic cancer cell lines, consistent with reported literature [8, 30]. Collectively, these results illustrate that in order for this targeted combination of agents to be effective, sensitive cells must exhibit both mutant *KRAS*-driven Gln dependence and *NQO1* expression. The latter is a feature of most, but not all, mutant *KRAS*-transformed cancer types.

### *GLS1* inhibition attenuates anti-oxidant defenses and increases susceptibility to ß-lap-induced DNA damage

**Fig. 4:**
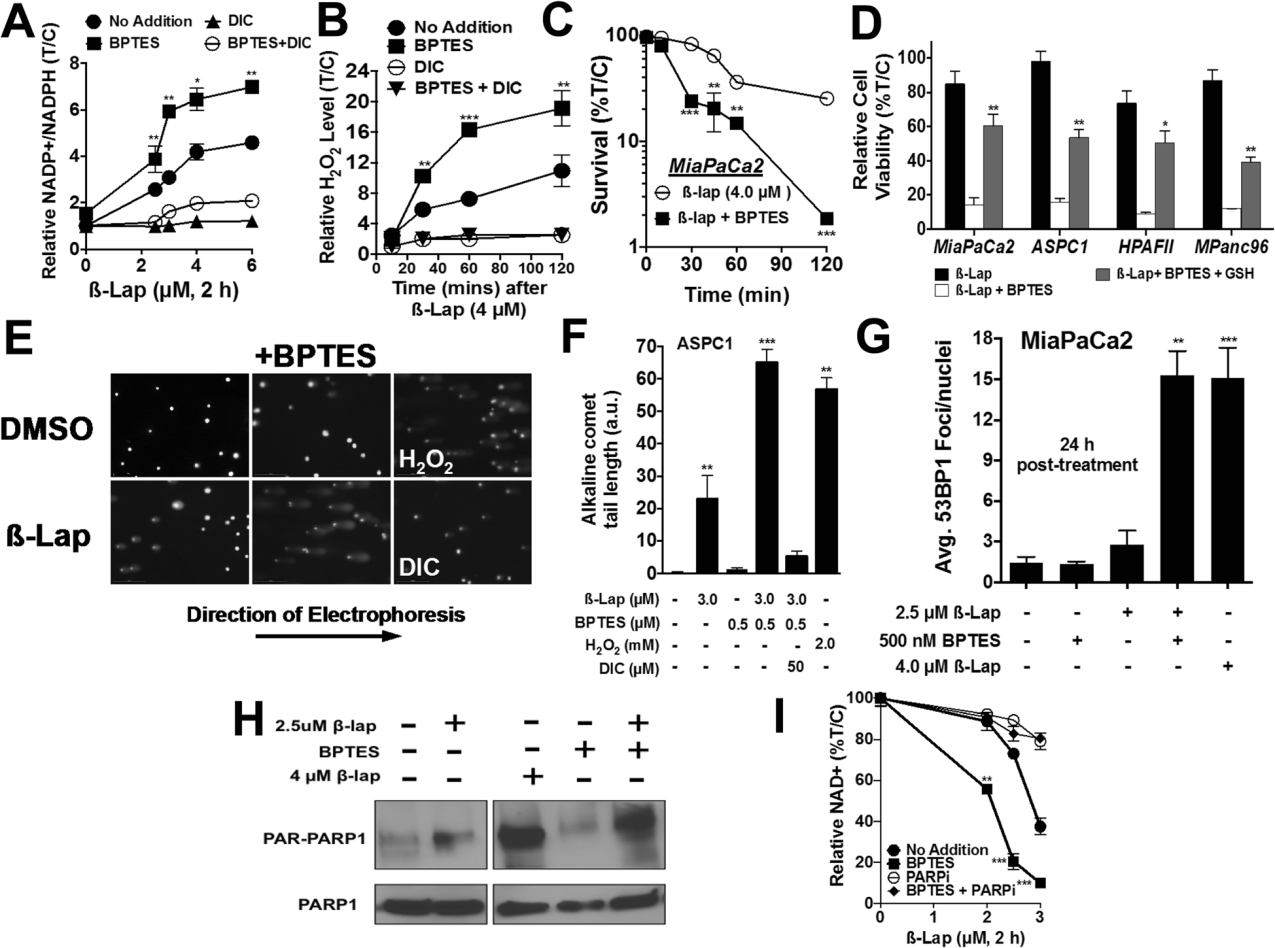
*GLS1* inhibition decreases anti-oxidant defenses and increases susceptibility to ß-lap-induced DNA damage. **a** Relative NADP+ to NADPH ratio in MiaPaCa2 cells pre-treated with BPTES for 48 h (500 nM) and then treated with ß-lap for 2 h. NADP+ and NADPH levels were measured immediately after 2 h treatment. **b** Relative extracellular H_2_O_2_ was measured through luminescence assay from the media of 4 μM ß-lap ± DIC, ±BPTES-treated MiaPaCa2 over a time-frame of 120 min, ±SE from sextuplicate samples. **c** Clonogenic survival of MiaPaCa2 pre-treated ± 500 nM BPTES for 48 h followed by the addition of ß-lap for various incubation times. Data represent survival means ± SE from quadruplicate samples. **d** MiaPaCa2, ASPC1, HPAFII, and MPanc96 pre-treated with ±500 nM BPTES and ±GSH reduced ethyl ester for 48 h followed by the addition of ß-lap for 2 h. **e**, **f** Alkaline comet assay of ASPC1 PDA cell lines pre-treated with ±500 nM BPTES followed by 2 h of ß-lap. **g** Average 53BP1 foci 24 h post treatment in MiaPaCa2. **h** Western blot for PAR formation with indicated treatment after 15 min of ß-lap exposure. **i** Relative NAD+ levels ±BPTES with various doses of ß-lap after 2 h of treatment. All results were compared using Student’s *t* tests. *p < 0.05; **p < 0.01; ***p < 0*.001*

We observed a dose-dependent increase in NADP+/NADPH ratios, a proxy for the cell’s oxidative state, in MiaPaCa2 cells exposed to ß-lap alone, reaching fourfold higher levels versus baseline found in untreated cells (Fig. 4a). With BPTES pre-treatment, we noted a sevenfold increase in NADP+/NADPH ratios in ß-lap-exposed cells versus baseline levels in untreated MiaPaCa2 cells (Fig. 4a) and an ~twofold higher level than in cells exposed to ß-lap alone. BPTES pre-treatment (500 nM, 48 h) also significantly lowered reduced glutathione (GSH) levels in MiaPaCa2 cells compared to DMSO vehicle alone (>twofold; Additional file 3: Figure S3A), and extracellular H_2_O_2_ production was dramatically increased after BPTES plus ß-lap treatment in a time- and dose-dependent manner (Fig. 4b, Additional file 3: Figure S3B). Additionally, total intracellular ROS levels were dramatically increased after BPTES plus ß-lap treatment (Additional file 3: Figure S3C). Consistent with the kinetics of H_2_O_2_ production, we observed a decrease in the minimum time to death [31, 32] for ß-lap-induced lethality in MiaPaCa2 with BPTES pre-treatment (Fig. 4c). This effect on clonogenic survival could be rescued with the anti-oxidant reduced diethyl-ester GSH in ß-lap-exposed MiaPaCa2, ASPC1, HPAFII, and MPanc96 cells after 48 h pre-treatment with BPTES plus 2 h with ß-lap (Fig. 4d).

Pre-treatment with BPTES followed by exposure to ß-lap synergistically increased total DNA lesions in ASPC1 cells, as assessed by alkaline comet assay immediately after 2-h treatment, and DNA double-strand break (DSB) formation in MiaPaCa2 cells, monitored by 53BP1 foci formation 24 h after treatment (Figs. 4e–g). Furthermore, pre-treatment with BPTES followed by treatment with ß-lap dramatically increased *PARP* hyperactivation noted by concomitant NAD+ depletion and PAR formation, which was blocked with the addition of the *PARP* inhibitor, Rucaparib (AG014699), in MiaPaCa2 cells compared to either treatment alone (Fig. 4h, i). These data indicate that PDA cells are reliant on glutamine for redox balance and that the disrupted redox state enhances ß-lap-induced DNA damage and *PARP*-driven metabolic catastrophe.

### *GLS1* inhibition sensitizes PDA to ß-lap in vivo

**Fig. 5:**
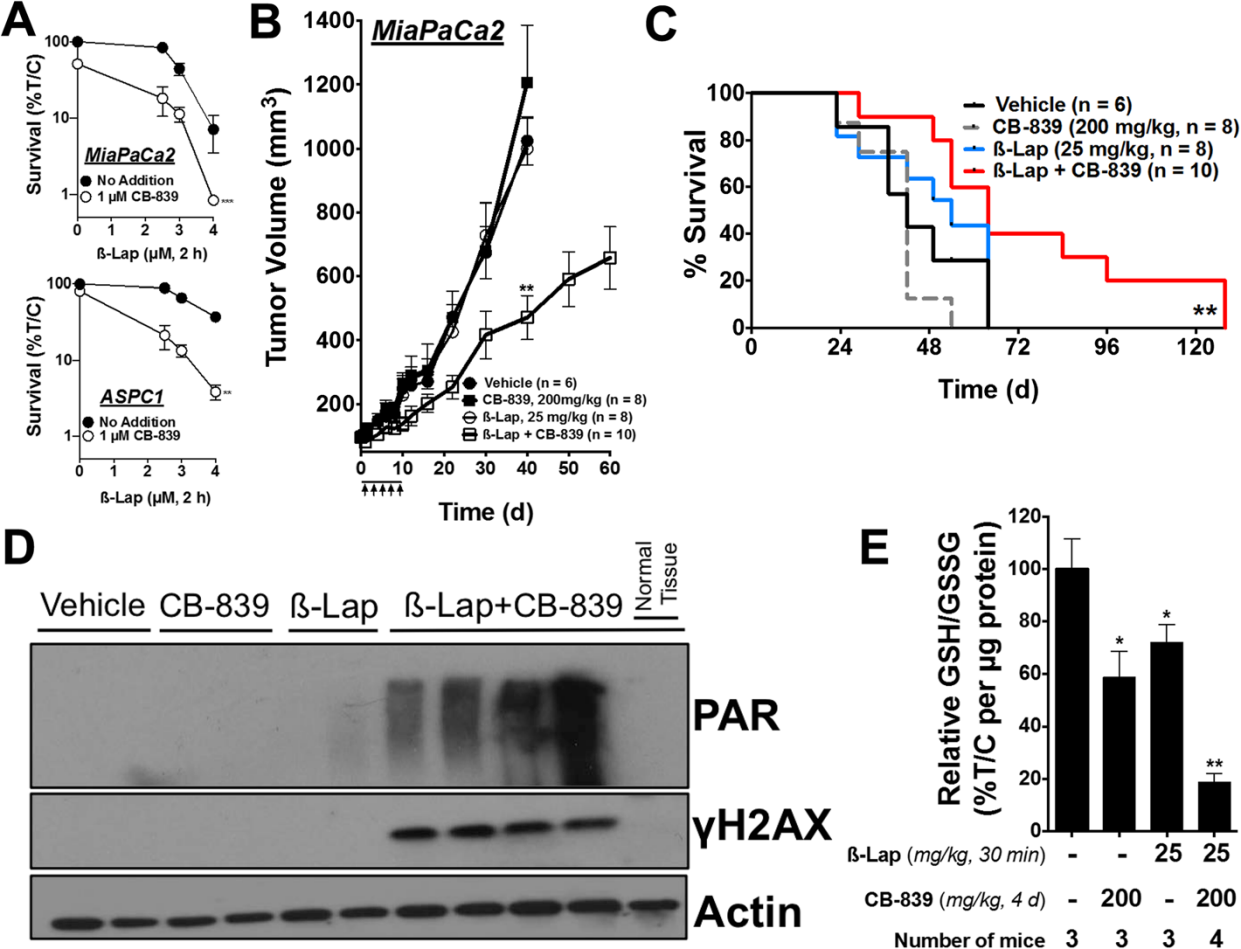
*GLS1* inhibition sensitizes pancreatic cancer to ß-lap in vivo. **a** Clonogenic survival of MiaPaCa2 and ASPC1 cells pre-treated with 1 μM CB-839 for 48 h followed by 2 h of ß-lap dose response. **b** Subcutaneous tumors grown from MiaPaCa2 cells in nude mice were allowed to reach a volume of 100 mm^3^, after which, the mice were treated every other day with vehicle (HPßCD, *n* = 6), sub-efficacious dose of CB-839 (oral gavage, 200 mg/kg, twice daily for 10 days, *n* = 8), sub-efficacious dose of ß-lap (IV, 25 mg/kg, *n* = 8) or sub-efficacious doses of CB-839 and ß-lap (*n* = 10) for a total of five doses (*arrows*). Tumor growth was monitored until tumors reached 1000 mm^3^. *Error bars*, SEM. **c** Survival of tumor-bearing mice represented as a Kaplan–Meier plot. The mice were sacrificed when tumors reached 1000 mm^3^. Statistically analyzed with the log-rank test for trend. **d** Western blot of PAR and γH2AX from a set of tumors harvested 30 min after treatment with ß-lap ± CB-839 (4-day treatment), *n* = 3 tumors per group, same doses as above. **e** Relative GSH/GSSG ratio in treated groups normalized to microgram of tumor protein. Unless stated otherwise, all results were compared using Student’s *t* tests. *p < 0.05; **p < 0.01; ***p < 0*.001*

To determine whether pharmacologic inhibition of *GLS1* in combination with ß-lap would lead to synergistic inhibition of PDA tumor growth in vivo, we utilized the clinical formulation of ß-lap (ARQ761), hydroxypropyl beta cyclodextrin travel (HPßCD)-ß-lap, and the orally available *GLS1* inhibitor, CB-839, provided by Calithera Biosciences. Both compounds are in separate phase I/II clinical trials for a variety of cancer types (NCT01502800, NCT02071862, NCT02071888, and NCT02071927) [11]. CB-839 was employed for these studies because BPTES has poor metabolic stability and low solubility in vivo [11]. First*,* we confirmed that CB-839 pre-treatment, like BPTES, also sensitized PDA cell lines in vitro to ß-lap in the MiaPaCa2 and ASPC1 lines (Fig. 5a). Next, we generated subcutaneous tumors from human MiaPaCa2 cells injected into the right hind limb in Nu/Nu female athymic mice and allowed the tumors to grow to a volume of 100 mm^3^ before beginning treatment. The mice were sacrificed when tumor volumes reached 1000 mm^3^, as per the Institutional Animal Care and Use Committee (IACUC) regulations.

The animals received either vehicle (i.e., HPßCD) or CB-839 (200 mg/kg) by oral gavage twice a day for 10 days, with or without a sub-efficacious dose of ß-lap (25 mg/kg) administrated intravenously (IV) every other day (Fig. 5b, arrows) [33]. After only one regimen of treatment (10 days), we found that the mice treated with CB-839 plus ß-lap displayed significantly delayed tumor growth compared to either agent alone through day 60. Importantly, we noted that neither CB-839 (200 mg/kg) nor ß-lap (25 mg/kg) administered alone significantly altered tumor growth (Fig. 5b). Individual tumors are presented in waterfall plots in Additional file 4: Figure S4A. Notably, combination treatment did not decrease mouse weights when compared to vehicle-treated mice (Additional file 4: Figure S4B). Long- or short-term toxicities, including hemolysis and methemoglobinemia, were not observed.

The mice were sacrificed when their original body weights dropped by one third, tumor volumes exceeded 1000 mm^3^, or when tumors began to ulcerate or impede normal motion. Kaplan–Meier curves showed a significant anti-tumor effect of the drug combination (Fig. 5c). The treatment with a sub-efficacious dose of ß-lap (25 mg/kg) resulted in a median survival of 49 days, while the vehicle-treated group displayed a median survival time of 39.5 days (Fig. 5c). The treatment with a sub-efficacious dose of CB-839 resulted in a median survival time of 42 days. The treatment with the ß-lap plus CB-839 combination resulted in a median survival of 64 days, significantly extending median survival by 24.5 days compared to the vehicle (HPßCD)-treated group (Fig. 5c).

Importantly, to ensure that the anti-tumor efficacy we observed was due to on-target effects of both drugs and by the same mechanism of action observed in vitro, we analyzed the pharmacodynamics profile of each agent alone and in combination. Briefly, the MiaPaCa2 tumor-bearing mice received either vehicle (HPßCD) alone or 200 mg/kg CB-839 by oral gavage twice a day for 4 days, with or without a single sub-efficacious dose of ß-lap at 25 mg/kg IV on day 4. After the last dose of CB-839 and 30 min after ß-lap injection, the mice were sacrificed and the tumor tissue was harvested. Tumor glutamate levels were measured from multiple animals for each treatment condition (Additional file 4: Figure S4C). CB-839 and CB-839 plus ß-lap-treated mice displayed significantly lower overall tumor glutamate levels when compared to vehicle or ß-lap alone treatments, consistent with *GLS1* inhibition in vivo (Additional file 4: Figure S4C).

We then immunoblotted tumor tissue lysates for PAR polymer formation as a proxy for *PARP* hyperactivity and γH2AX to assess DNA DSBs (Fig. 5d, see quantification in Additional file 4: Figure S4D) [33]. Consistent with our results in vitro, we found that the mice treated with the combination of ß-lap plus CB-839 displayed dramatically increased PAR and γH2AX formation relative to all other groups (Fig. 5d). Additionally, we harvested liver tissue (“normal tissue” in Fig. 5d) from the mice after exposure to the combination-treated mice and found no evidence of PAR or γH2AX formation (Fig. 5d), consistent with a lack of response to ß-lap in normal tissues [24, 33]. Notably, the liver contains the highest levels of *NQO1* in the mouse (not in human livers) and is used as a surrogate for normal tissue responses to *NQO*1-bioactivatable drugs.

Next, to determine the redox status of tumors after treatment, we measured the GSH to glutathione disulfide (GSSG) ratio of tumor lysates after vehicle alone or CB-839, with or without 30-min ß-lap treatment. Interestingly, we found that the GSH to GSSG ratios were significantly decreased in the single-arm CB-839 or ß-lap-treated mice (Fig. 5e). Moreover, the combination treatment resulted in an even greater decrease in GSH, monitored by the GSH to GSSG ratio (Fig. 5e). Taking our pharmacodynamics observations and anti-tumor studies together, these data demonstrate that modulating glutamine metabolism in PDA in vivo results in a significantly decreased anti-oxidant defense state. As a result, the drug combination significantly sensitized *NQO1*-expressing tumors, but not associated normal tissue, to ROS induction from ß-lap leading to DNA damage, *PARP* hyperactivation, and tumor-selective death.

## Discussion

Tumor cells display unique metabolic alterations that impact the biological behavior of the tumor and have therapeutic implications [34, 35]. A well-documented example of such an alteration is the increased utilization of glutamine in a variety of human tumors, including lung, prostate, lymphoma, and PDA [8, 15, 36, 37]. Glutamine has pleiotropic roles in the cell that include the regulation of autophagy, signal transduction, anabolic growth, and redox balance [9]. In PDA, mutant *KRAS* promotes the reprogramming of glutamine metabolism through a *GOT1-* and *GOT2*-mediated transamination pathway instead of the pathway catalyzed by *GLUD1* [8]. This transamination pathway is necessary to maintain redox balance in PDA tumors through maintenance of reduced NADPH and GSH levels (Fig. 1a) [2, 8, 9]. While this understanding provides insight into PDA metabolism, how to most appropriately exploit this metabolic vulnerability had remained to be determined. Previous work revealed that targeting this pathway alone resulted in cytostasis, driving compensatory metabolic resistance pathways in the tumor. In addition, potential systemic toxicities remain to be determined.

Genes involved in the glutamine-dependent transamination pathway (*GLS1*, *GOT1/2*, *ME1*) and *NQO1*, but not *GLUD1*, are highly expressed in PDA relative to other cancers (Fig. 1b). Clinical PDA cases expressing a high *GOT1* to *GLUD1* or *GOT2* to *GLUD1* ratio in the tumor have worse outcomes (Fig. 1c, Additional file 1: Figure S1B). Here, we found that genetic or pharmacological inhibition of this pathway sensitizes PDA to the *NQO1*-bioactivatable drug, ß-lap. Addition of ß-lap in combination with *GLS1* inhibition resulted in enhanced tumor-cell specificity and efficacy in PDA cell lines, not typically found with *GLS1* inhibition alone. Consistent with prior reports of differential usage of glutamine in mutant *KRAS* versus wild-type *KRAS* cancers [4, 8, 30, 38], we found that mutant *KRAS* cell lines, but not wild-type *KRAS* PDA, lung, and breast cancer, were sensitized to ß-lap upon pre-treatment with BPTES. These data suggest that mutant, compared to wild-type, *KRAS* cancers may also rely on glutamine to maintain redox balance, though this may not necessarily involve the transamination pathway discussed. Future studies to elucidate this mechanism will be needed.

When considering the ROS burst generated from *NQO1*-bioactivatable drugs, as evidenced by ß-lap’s H_2_O_2_ production profile, cells require an equally robust anti-oxidant response to suppress ROS accumulation. Thus, one can imagine that a competition between the rates of engagement of the anti-oxidant machinery and the production of ROS determines the fate of cancer cells within a tumor following *NQO1-*bioactivatable drug treatment. Notably, we previously reported that catalase (an essential enzyme for the detoxification of 2H_2_O_2_ to 2H_2_O + O_2_) expression was significantly downregulated in tumor tissue relative to normal tissue, and that the *NQO1* to catalase ratio is markedly increased in tumor tissue. Moreover, addition of PEGylated catalase significantly protected breast cancer cell lines from ß-lap-induced lethality [25]. To maintain redox balance, PDA may utilize glutamine metabolism to compensate for this decrease in catalase. Indeed, addition of BPTES to *NQO1*-overexpressing cancer cells decreased NADPH and GSH anti-oxidant defenses, which would otherwise counteract the ß-lap-induced ROS burst and thus lead to enhanced DNA damage, *PARP*-driven NAD+ and ATP depletion, and metabolic catastrophe. Mechanistically, this occurred in a near-identical manner to the caspase-independent, μ-calpain-mediated cell death pathway induced by lethal doses of ß-lap alone [39], only at lower doses, which act to augment the therapeutic window for ß-lap.

For our in vivo studies, we utilized the *GLS1* inhibitor CB-839, as this compound has far greater solubility and stability in animals than BPTES [11]. A single regimen of CB-839 plus ß-lap in the tumor-bearing mice slowed tumor progression and significantly improved survival compared to the animals treated with either agent alone. Pharmacodynamics analysis revealed that the mechanism of synergy with combination treatment in vivo was consistent with the in vitro results. In sum, these results are promising but preliminary in nature, given their reliance on subcutaneous PDA cell line-derived xenografts, as these models may overestimate the efficacy of therapeutic regimens [40]. Future work will test combination treatment regimens in genetically engineered PDA mouse models [41]. We should note, however, that subcutaneous xenograft models do represent a challenging microenvironment for pancreatic cancer studies due to the hypoxic environment and limited blood supply.

## Conclusions

Novel therapies are desperately needed for patients with PDA. By combining *GLS1* inhibition (e.g., CB-839) and *NQO1*-bioactivatable drugs (e.g., β-lap, ARQ761), we exploit the reliance of PDA on glutamine for redox balance, as well as the tumor-selective overexpression of *NQO1* through the use of a unique agent that is bioactivated to induce cell death. Combination treatment with *GLS1* inhibitors plus ß-lap addresses limitations associated with either agent alone. Namely, this combination is expected to enhance efficacy at well-tolerated doses of these drugs; the concentrations used in this study are relevant to those that are achievable in patients (NCT01502800, NCT02071862, NCT02071888, and NCT02071927) [11, 42]. Furthermore, we noted that CB-839 plus ß-lap combination-treated animals did not display increased cytotoxicity in normal liver tissue, based on a lack of PAR and DNA damage formation. These results reinforce the necessity for *NQO1* expression to achieve cell death. Collectively, these findings illustrate a rational combination strategy to target PDA dependence on glutamine, by *GLS1* inhibition, in combination with an *NQO1*-bioactivatable drug and provide the proof-of-concept validation to warrant analysis in a larger pre-clinical context.

## Methods

### Cell culture

A549, MiaPaCa2, DAN-G, SW1990, MPanc96, SUIT2-013-NQO1+/−, HPAFII, ASPC1, PL45, H596, Hs766T, BxPC3, MBA-MD-231+/−, and MCF7 cell lines were obtained from ATCC, tested for mycoplasma contamination and grown in complete Dulbecco's Modified Eagle's Medium (DMEM) with 10 % fetal bovine serum (FBS). H2122 and H661 cell lines were a generous gift from Dr. John Minna at UTSW. SUIT2-013 and MDA-MB-231 NQO1 deficient and proficient cell lines were generated as previously described [24]. DMEM (glutamine free) was purchased from Sigma-Aldrich. FBS was purchased from Fischer Scientific. Glutamine, GSH-reduced ethyl ester, OAA, and dimethyl malate were purchased from Sigma. IMR90 cells lines were grown in MEM with 10 % FBS. All cells were incubated at 37° with 5 % CO_2_.

### Reagents and chemicals

ß-Lap was synthesized, purified, and prepared at 50 mM stock solution as previously described [18]. BPTES and DIC were purchased from Sigma-Aldrich (St Louis, MO). CB-839 was kindly provided by Calithera Biosciences (San Francisco, CA).

### Survival assays

For clonogenic survival assays, the cells were plated in 10-cm plates at 2 × 10^5^ cells per plate and pre-treated with ±BPTES for 48 h, then treated with the appropriate ß-lap doses for 2 h. The cells were then trypsinized and plated onto six-well plates at 100, 500, or 1000 cells per well in 2 mL of complete media. After 7 days of proliferation, the plates were washed in PBS and colonies were fixed in methanol/crystal violet; all clonogenic assay results are represented as % Survival (treated/control, T/C). Cell viability was also assessed using the CellTiter-Glo assay (Promega). The cells were plated and pre-treated with ±BPTES as described above. The cells were then plated at 6 × 10^4^ cells per well in a 96-well plate in 100 μL of media. The following day, the cells were treated with the appropriate doses of ß-lap for 2 h, and then ATP levels were checked 48 h after treatment; cell viability is represented as Relative Cell Viability % (T/C). Relative cell survival was also assessed using 7-day DNA assays as previously described and reported as Relative Survival % (T/C) [43]. Results are reported as means ± standard error (SE) from at least three  independent experiments done in sextuplicate. This dose of ß-lap (4 μM, 2 h) typically yields 80–90 % lethality in MiaPaCa2 cell lines across these assays.

### Glutathione, NAD(P)H, H_2_O_2,_ and total ROS assays

The following assays were purchased from Promega: GSH/GSSG-Glo, NAD/NADH-Glo, NADP/NADPH-Glo, and ROS-Glo and were used as instructed by the manufacturer. The total intracellular ROS levels were detected using the cell-permeable fluorogenic probe CellROX® (Molecular Probes) that emits red fluorescence upon oxidation by ROS. Briefly, the cells were incubated with 5 μM CellROX® and ß-lap for 30 min at 37 °C followed by fixation in 4 % paraformaldehyde. ROS generation was analyzed by flow cytometry.

### siRNA knockdown

siRNA was transiently transfected into the cells using Opti-MEM and Lipofectamine RNAiMAX for 48 h (Life Technologies) for the following enzymes: GLS1, ON-TARGET plus GLS1 SMARTpool; GLS1, 5′-GAUGGAUUGUUGUAAUGGU-3′; GLUD1, 5′-CUCACUAUCCUCUUCACAU-3′; GOT1, 5′CUCUAACCCUGAGCUCUUU-3′; ME1, 5′-GACACUUAGAUUAAGAUUU-3′; and GOT2, 5′GCCUUUAAGAGGGACACCA-3′. All siRNAs were purchased from Sigma. After 48 h of incubation, the cells were detached with trypsin/EDTA (Life Technologies) and seeded for treatment assays or lysed for analysis of knockdown efficiency. Growing cells in Opti-MEM and Lipofectamine for 48 h increased their sensitivity to ß-lap in the siScr group alone.

### Western blot

Western blots were carried out as previously described [25]. Primary antibodies for protein detection included: GLS1 (ab93434, Abcam), PARP1 (F-2, Santa Cruz), PAR (Trevigen, Gaithersburg, MD), Actin (C-2, Santa Cruz), small subunit calpain (EPR3324, Abcam), and 53BP1 (Bethyl Laboratories).

### Human xenograft anti-tumor, pharmacodynamics assays

The athymic Nu/Nu nude female mice (18–20 g) were commercially obtained (Harlan). Human xenografts were generated by injecting 2 × 10^6^ MiaPaCa2 cells subcutaneously in PBS/Matrigel into 6-week-old mice. The tumors were measured at indicated times with digital calipers (Fisher Scientific), and tumor volumes calculated (length × width^2^ × 0.5). The treatments were initiated when subcutaneous tumors reached an average size of ≥100 mm^3^. The mice were treated with CB-839 by oral gavage, ß-lap (as HPßCD-ß-lap) by IV (retro-orbital) or both or with vehicle (HPßCD; 1:9, v/v; Sigma-Aldrich) as a control. The treatment regimens consisted of a total of five doses of ß-lap, with one dose being administered every other day for 10 days. CB-839 was administered twice a day, everyday for 10 days. The mice bearing subcutaneous tumors were treated with nontoxic doses (200 mg/kg) of CB-839, with or without a sub-efficacious dose of HPßCD-ß-lap (25 mg/kg). The mice bearing subcutaneous tumors were sacrificed when tumors reached >1000 mm^3^ as per IACUC. Overall survival was assessed through Kaplan–Meier curves using the log-rank test for trend for statistical significance. The mice were weighed three times per week during and after the drug treatment period with no toxicities observed. All animal studies were performed in accordance under protocols approved by the Institutional Animal Care and Use Committee of UT Southwestern Medical Center.

### Statistics

All graphs are plotted as mean with error bars denoting standard deviation. Regression analysis, ANOVA, and two-tailed Student *t* tests for statistical significance with Holm/Sidak multiple comparison correction were performed in GraphPad Prism 6. *p* values were expressed throughout by *p < 0.05, **p < 0.01, ***p < 0.001, and ****p < 0.0001.

## Additional files

Additional file 1: Figure S1. ß-Lap mechanism and *GOT2* to *GLUD1* Kaplan–Meier curve. (A) ß-lap mechanism of action. In the cytosol of cancer cells, one equivalent of β-lap undergoes a futile redox cycle with NQO1 to produce ~120 equivalents of H_2_O_2_, in 2–5 min (depending on cancer cell), which results in DNA damage, PARP1 hyperactivation, NAD+ and ATP depletion, and ultimately caspase-independent apoptosis (a form of cell death known as programmed necrosis). NAD, nicotinamide adenine dinucleotide; NADPH, nicotinamide adenine dinucleotide phosphate (reduced); NQO1, NADPH:quinone oxidoreductase; PARP1, poly(ADP ribose) polymerase 1. (B) *GOT2* to *GLUD1* Kaplan–Meier curve, *n* = 45 patients. Additional file 2: Figure S2. BPTES sensitivity. (A) Growth inhibition in MiaPaCa2 cells, as monitored by loss of ATP, to short-term (48 h) or long-term (96 h) BPTES treatments. Additional file 3: Figure S3. Combination treatment leads to increased ROS by depleting reduced glutathione. (A) Relative GSH levels ±500 nM BPTES pre-treatment followed by ß-lap treatment. (B) Relative H_2_O_2_ production with ±500 nM BPTES pre-treatment followed by ß-lap treatment. (C) CellROX® quantification of total ROS at 15 min of ±4 μM ß-lap, ±DIC, and ±BPTES (48-h pre-treatment). Additional file 4: Figure S4. Assessment of drug effects on PDA tumors and animals. (A) Change in tumor volumes per animal at day 22 compared to day 1. (B) Mouse weights during treatment for vehicle (*n* = 6), CB-839 (*n* = 8), ß-lap (*n* = 8) and ß-lap plus CB-839 (*n* = 10). (C) Tumor glutamate levels, *n* = 3 mice per group. (D) Quantification of relative PAR and γH2AX from tumor tissue lysate according to the western blot in Fig. 5d.

## Abbreviations

ADP: adenosine diphosphate
ATP: adenosine triphosphate
DIC: dicoumarol
GEMM: genetically modified mouse model
GLS1: mitochondrial glutaminase 1
GLUD1: glutamate dehydrogenase 1
GOT1: cytoplasmic glutamate oxaloacetate transaminase 1
GOT2: mitochondrial glutamate oxaloacetate transaminase 2
GSH: glutathione (reduced)
KRAS: Kirsten rat sarcoma viral oncogene homolog
MDH1: cytosolic malate dehydrogenase 1
ME1: malic enzyme 1
NAD: nicotinamide adenine dinucleotide
NADPH: nicotinamide adenine dinucleotide phosphate (reduced)
NQO1: NADPH:quinone oxidoreductase
PAR: poly(ADP ribose)
PARP: poly(ADP ribose) polymerase
PD: pharmacodynamics
PDA: pancreatic ductal adenocarcinoma
PK: pharmacokinetics
ROS: reactive oxygen species.
ß-lap: ß-lapachone
