# Supplementary figures and images for: Targeting glutamine metabolism sensitizes pancreatic cancer to PARP-driven metabolic catastrophe induced by ß-lapachone
Source: Cancer Metab. 2015 Oct 12;3:12. doi: 10.1186/s40170-015-0137-1 (PMC4601138; doi:10.1186/s40170-015-0137-1)

## Slide 1
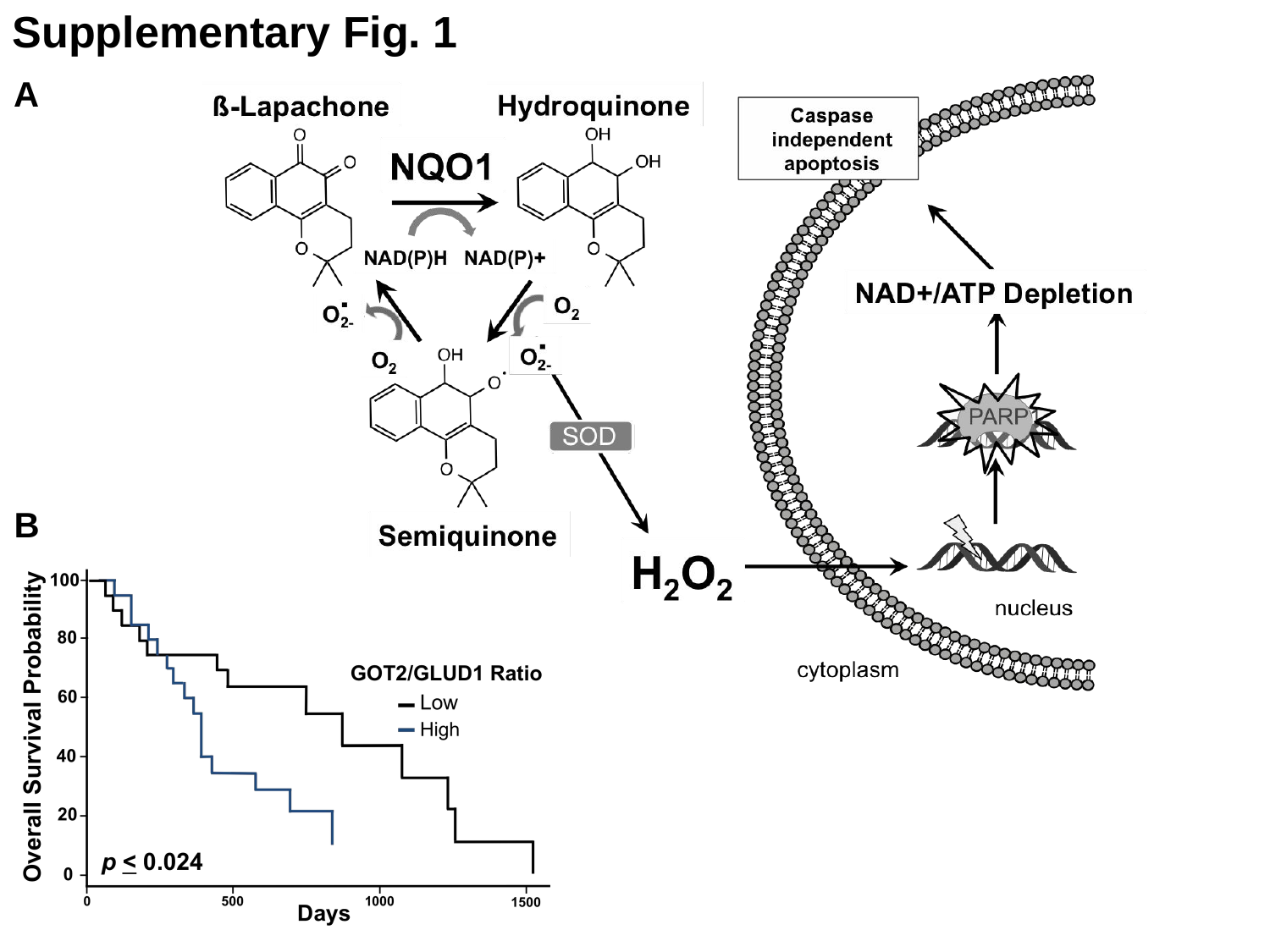

Supplementary Fig. 1
A
B

Supplement: Additional file 1: Figure S1. — ß-Lap mechanism and GOT2 to GLUD1 Kaplan–Meier curve. (A) ß-lap mechanism of action. In the cytosol of cancer cells, one equivalent of β-lap undergoes a futile redox cycle with NQO1 to produce ~120 equivalents of H2O2, in 2–5 min (depending on cancer cell), which results in DNA damage, PARP1 hyperactivation, NAD+ and ATP depletion, and ultimately caspase-independent apoptosis (a form of cell death known as programmed necrosis). NAD, nicotinamide adenine dinucleotide; NADPH, nicotinamide adenine dinucleotide phosphate (reduced); NQO1, NADPH:quinone oxidoreductase; PARP1, poly(ADP ribose) polymerase 1. (B) GOT2 to GLUD1 Kaplan–Meier curve, n = 45 patients. [file 40170_2015_137_MOESM1_ESM.ppt]

## Slide 1
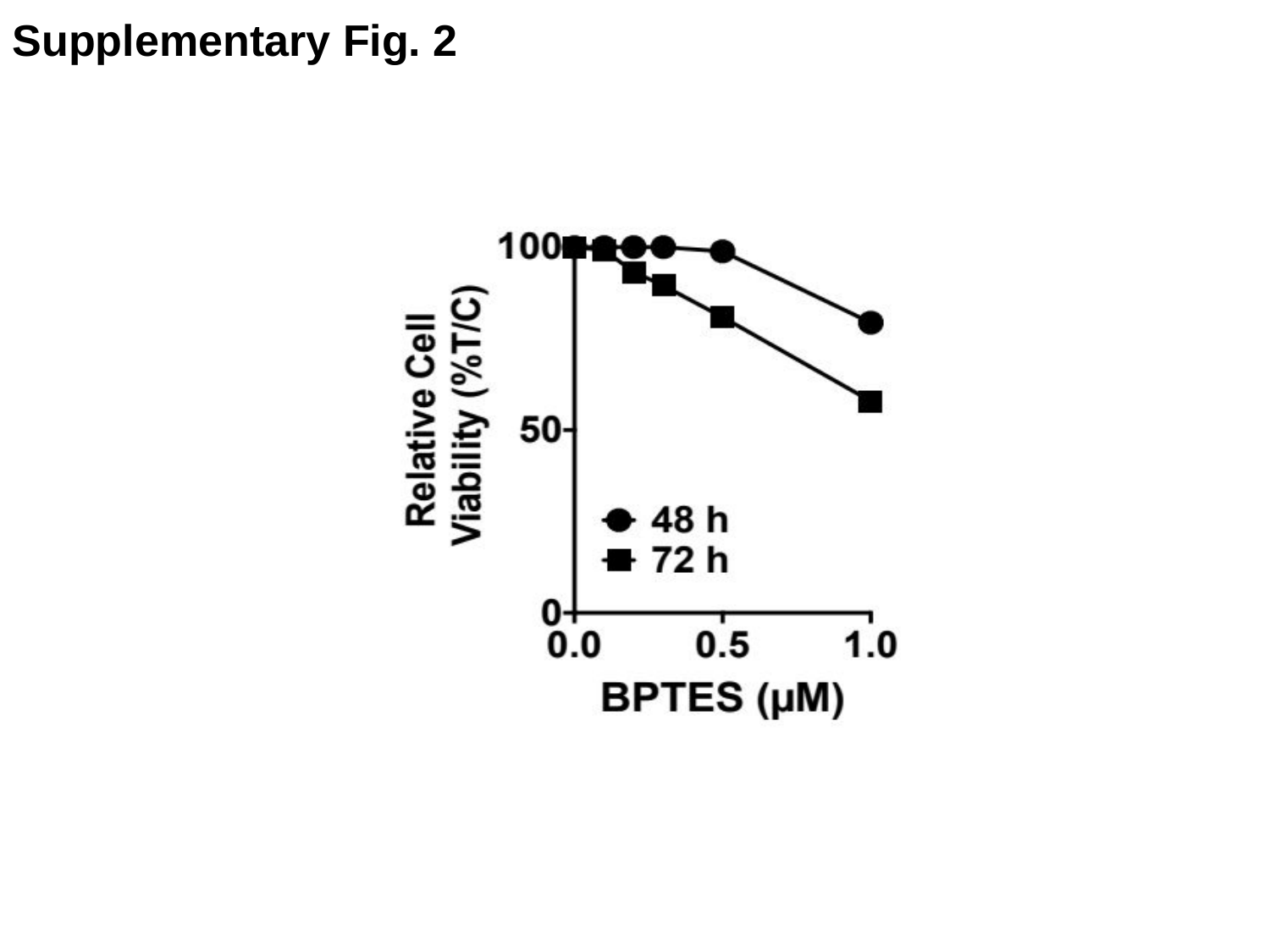

Supplementary Fig. 2

Supplement: Additional file 2: Figure S2. — BPTES sensitivity. (A) Growth inhibition in MiaPaCa2 cells, as monitored by loss of ATP, to short-term (48 h) or long-term (96 h) BPTES treatments. [file 40170_2015_137_MOESM2_ESM.ppt]

## Slide 1
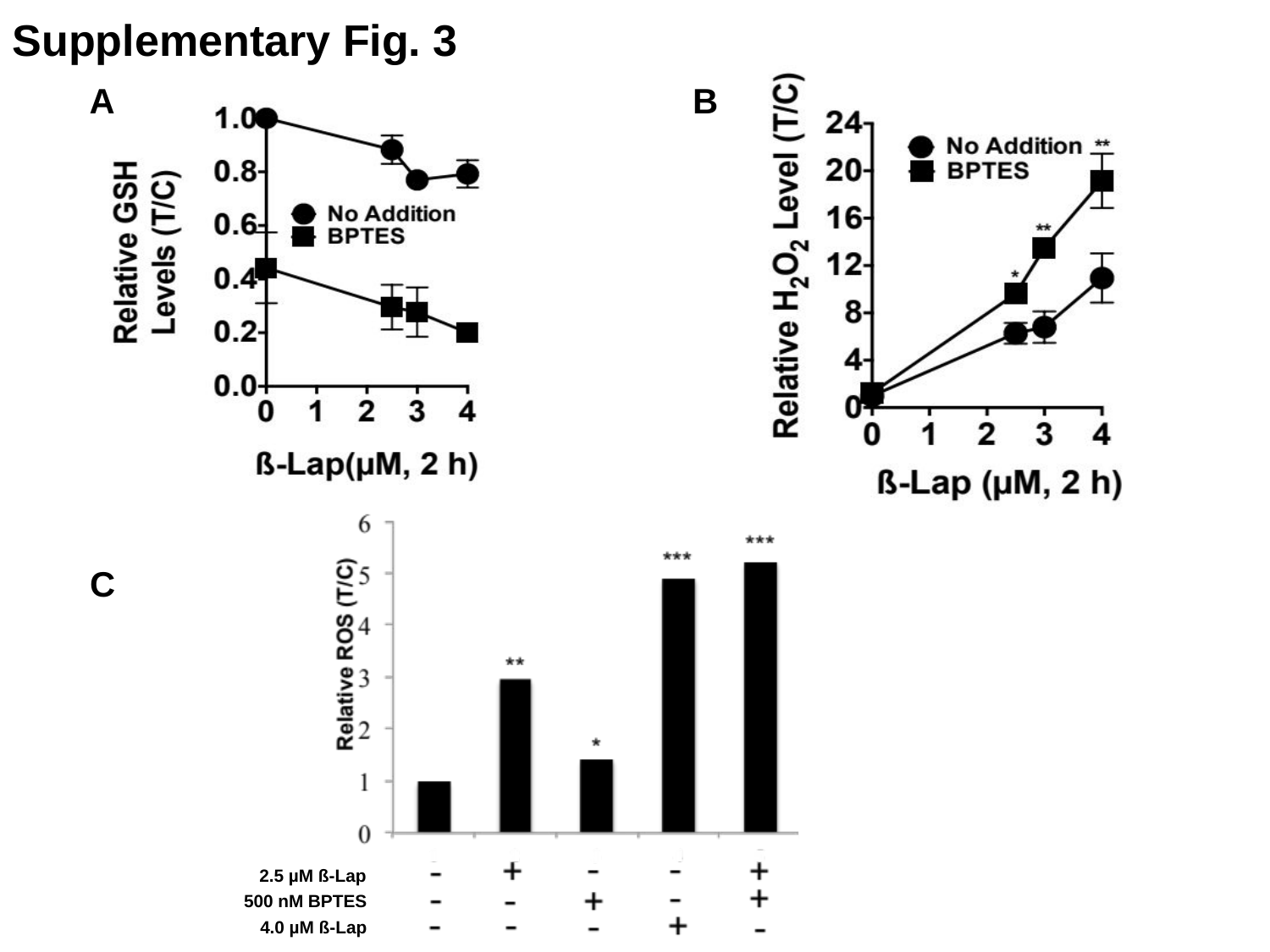

Supplementary Fig. 3
A
B
2.5 µM ß-Lap
500 nM BPTES
4.0 µM ß-Lap
C

Supplement: Additional file 3: Figure S3. — Combination treatment leads to increased ROS by depleting reduced glutathione. (A) Relative GSH levels ±500 nM BPTES pre-treatment followed by ß-lap treatment. (B) Relative H2O2 production with ±500 nM BPTES pre-treatment followed by ß-lap treatment. (C) CellROX® quantification of total ROS at 15 min of ±4 μM ß-lap, ±DIC, and ±BPTES (48-h pre-treatment). [file 40170_2015_137_MOESM3_ESM.ppt]

## Slide 1
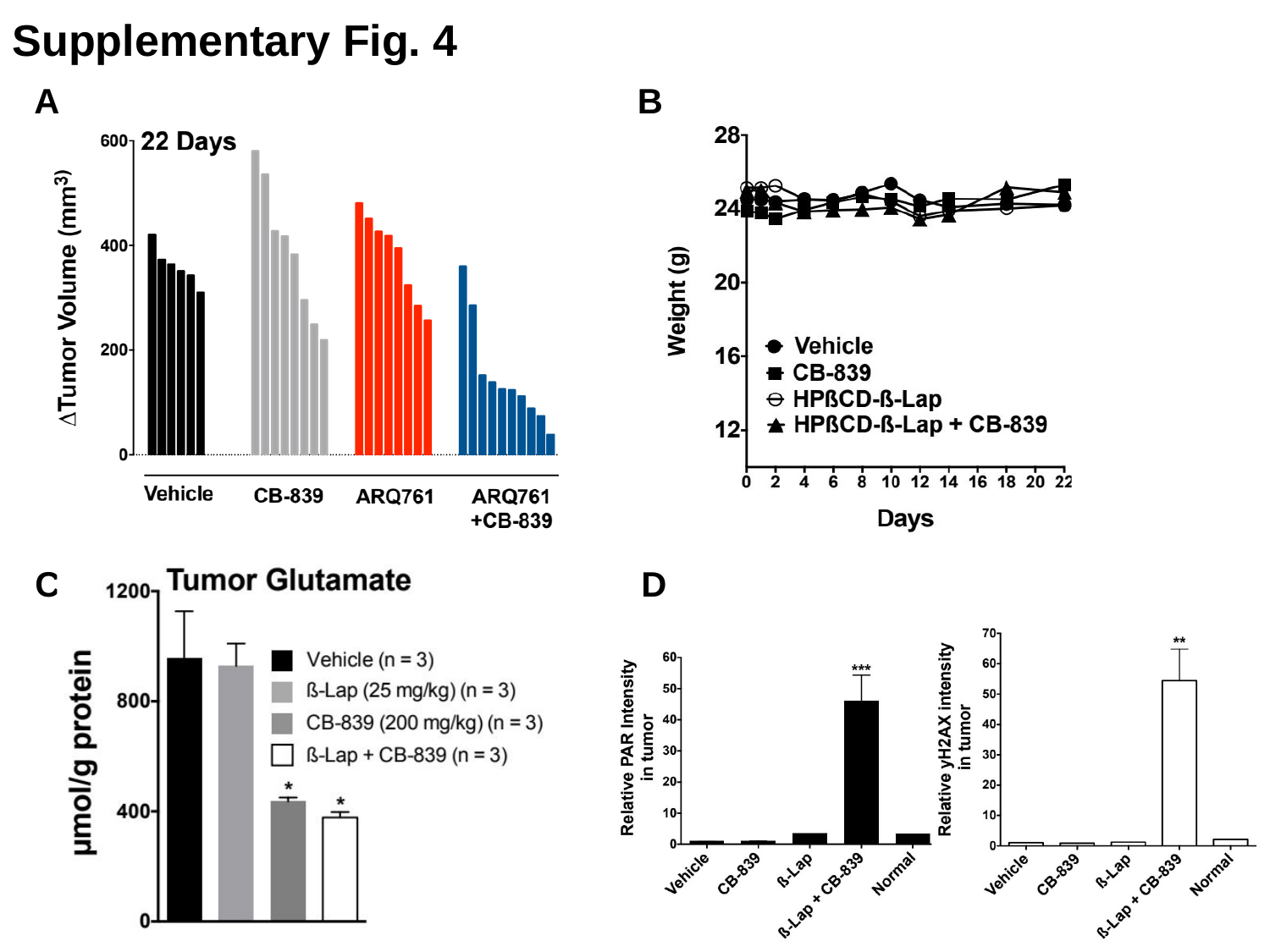

Supplementary Fig. 4
A
B
C
D

Supplement: Additional file 4: Figure S4. — Assessment of drug effects on PDA tumors and animals. (A) Change in tumor volumes per animal at day 22 compared to day 1. (B) Mouse weights during treatment for vehicle (n = 6), CB-839 (n = 8), ß-lap (n = 8) and ß-lap plus CB-839 (n = 10). (C) Tumor glutamate levels, n = 3 mice per group. (D) Quantification of relative PAR and γH2AX from tumor tissue lysate according to the western blot in Fig. 5d. [file 40170_2015_137_MOESM4_ESM.ppt]
